# Pioglitazone reduces cardiovascular events and dementia but increases bone fracture in elderly patients with type 2 diabetes mellitus: a national cohort study

**DOI:** 10.18632/aging.204643

**Published:** 2023-04-07

**Authors:** Chieh-Li Yen, Chao-Yi Wu, Chung-Ying Tsai, Cheng-Chia Lee, Yi-Jung Li, Wei-Sheng Peng, Jia-Rou Liu, Yuan-Chang Liu, Chang-Chyi Jenq, Huang-Yu Yang, Lai-Chu See

**Affiliations:** 1 Department of Nephrology, Kidney Research Institute, Chang Gung Memorial Hospital at Linkou, College of Medicine, Chang Gung University, Taoyuan City, Taiwan; 2 Department of Internal Medicine, Division of Rheumatology, Allergy and Immunology, Chang Gung Memorial Hospital at Linkou, Taoyuan City, Taiwan; 3 Department of Pediatrics, Division of Allergy, Asthma and Rheumatology, Chang Gung Memorial Hospital at Linkou, College of Medicine, Chang Gung University, Taoyuan City, Taiwan; 4 Department of Public Health, College of Medicine, Biostatistics Core Laboratory, Molecular Medicine Research Center, Chang Gung University, Taoyuan City, Taiwan; 5 Department of Medical Imaging and intervention, Chang Gung Memorial Hospital at Linkou, Taoyuan City, Taiwan; 6 Department of Health Policy and Management, Johns Hopkins University Bloomberg School of Public Health, Baltimore, MD 21205, USA

**Keywords:** cardiovascular disease, dementia, diabetes, elderly, pioglitazone

## Abstract

The prevalence of type 2 diabetes (T2DM) in elderly people has expanded rapidly. Considering cognitive impairment and being prone to hypoglycemia of the elder, the pros and cons of oral hypoglycemic agents (OHA) should be reassessed in this population. Pioglitazone might be appropriate for elderly DM patients because of its insulin-sensitizing effect and low risk of hypoglycemia.

By using Taiwan’s National Health Insurance Research Database, 191,937 types 2 diabetes patients aged ≥65 years under treatment between 2005 and 2013 were identified and further divided into two groups according to whether they received pioglitazone (pioglitazone group) or other OHAs (non-pioglitazone group) in the 3 months preceding their first outpatient visit date after 65 years of age, with a diagnosis of T2DM. Propensity score stabilization weight (PSSW) was used to balance the baseline characteristics. In results, the pioglitazone group (*n* = 17,388) exhibited a lower rate (per person-years) of major advanced cardiovascular events MACCE (2.76% vs. 3.03%, hazard ratio [HR]: 0.91, 95% confidence interval [CI]: 0.87–0.95), new- diagnosis dementia (1.32% vs. 1.46%, HR: 0.91, 95% CI: 0.84–0.98) but a higher rate of new-diagnosis bone fractures (5.37% vs. 4.47%, HR: 1.24, 95% CI: 1.19–1.28) than the non-pioglitazone group (*n* = 174,549). In conclusion, using pioglitazone may reduce the risks of MACCE and dementia but increases the probability of bone fractures in the elderly DM population.

## INTRODUCTION

Type 2 diabetes mellitus, a major threat to health, affects almost 10% of the global population [1]. Compared to the overall population with diabetes, the number of diabetes cases in elderly people has increased rapidly in recent decades [2]. Type 2 diabetes (T2DM) is a prevalent disorder in the elderly, with approximately one-quarter of people aged ≥ 65 with diabetes and an expected increase in rates of diabetes in the upcoming years [3]. In addition, the number of elderly diabetes patients is projected to reach 195.2 million by 2030 and 276.2 million by 2045 [4]. Because of the higher incidence of type 2 diabetes in the elderly, the aging of the global population is the most powerful driver of the diabetic epidemic [5]. Thus, type 2 diabetes in elderly patients has become an important issue due to observed population aging.

Several physical differences exist between elderly and younger patients with type 2 diabetes. First, the risk of malnutrition and skeletal muscle loss increases with advancing age [6]. Because of the important role of skeletal muscles in glucose metabolism, their loss causes frailty and risk of infection and leads to insulin resistance [7]. In turn, type 2 diabetes-associated insulin resistance is also involved in sarcopenia and frailty with aging [8, 9]. Second, dementia in elderly patients with diabetes is almost twice that of their age-matched non-diabetic counterparts [10]. A high rate of cognitive dysfunction in elderly patients increases the subsequent risk of hypoglycemia due to difficulties in performing complex self-care tasks or low early awareness of hypoglycemic symptoms [11]. In addition, recurrent hypoglycemia has been proven to cause further progression of dementia [12]. Third, due to decreased gluconeogenesis, defective counterregulatory mechanisms, and cognitive dysfunction, elderly diabetes patients are much more vulnerable to severe hypoglycemia [13]. Severe hypoglycemia is a life-threatening event in the elderly, which may lead to stroke, seizure, cognitive function decline, and increased risk of cardiovascular (CV) events or CV deaths in the following months [12, 14]. Considering physical alterations in elderly diabetes patients, the pros and cons of commonly used oral hypoglycemic agents (OHA) in the general population should be reassessed in elderly diabetes patients.

Pioglitazone, a thiazolidinedione (TZD), can improve systemic insulin sensitivity and effectively reduce blood sugar by binding to peroxisome proliferator-activated receptor gamma (PPAR-γ). In addition, pioglitazone is associated with a lower incidence of hypoglycemia and can be safely used in patients with chronic kidney disease (CKD) without the need for dose reduction [15]. These benefits make pioglitazone an attractive option for treating elderly patients with diabetes. However, safety issues associated with pioglitazone, including fluid and sodium retention and an increased risk of bone fractures, may lead to more severe consequences in elderly patients. Current evidence is less discussing the possible benefits and risks of using pioglitazone in elderly type 2 diabetes patients. Thus, by using Taiwan’s National Health Insurance Research Database (NHIRD), which is one of the largest healthcare datasets in the world, we designed this large-scale cohort study to compare the most interesting outcomes in elderly type 2 diabetes patients, namely all-cause mortality, CV death, infection-related death, dementia, heart failure, and bone fractures, between users and non-users of pioglitazone.

## METHODS

### Data source

The population of Taiwan is aging sharply, and the population aged 65 years and above has reached 16% of the total population of Taiwan in 2020, making Taiwan an appropriate candidate for analyzing different diabetes treatments in the elderly [16].

This research used data from the Taiwan National Health Insurance Research Database (NHIRD). In 1995, Taiwan launched the National Health Insurance (NHI) program, a national, single-payer compulsory health care program. Until 1997, this insurance system covered nearly 99.8% of Taiwan’s population (equivalent to 23.37 million) [17]. Comprehensive health care information on insured patients, including outpatient visits, medication prescriptions, diagnosis of diseases, management during hospitalization, procedure interventions, and registration of particular conditions, are available in the NHIRD. However, they do not contain laboratory data or examination reports. In the NHIRD, disease diagnoses were made based on the International Classification of Diseases, 9th Revision, Clinical Modification (ICD-9-CM) before 2015, and ICD-10-CM since 2016. In the NHIRD, personal identification (ID) or any information that could identify a specific person is scrambled before data are released for study purposes. This research was approved with a waiver of consent from the Institutional Review Board of Chang Gung Medical Foundation (approval number: 201900840B0).

### Study design

**Figure 1:**
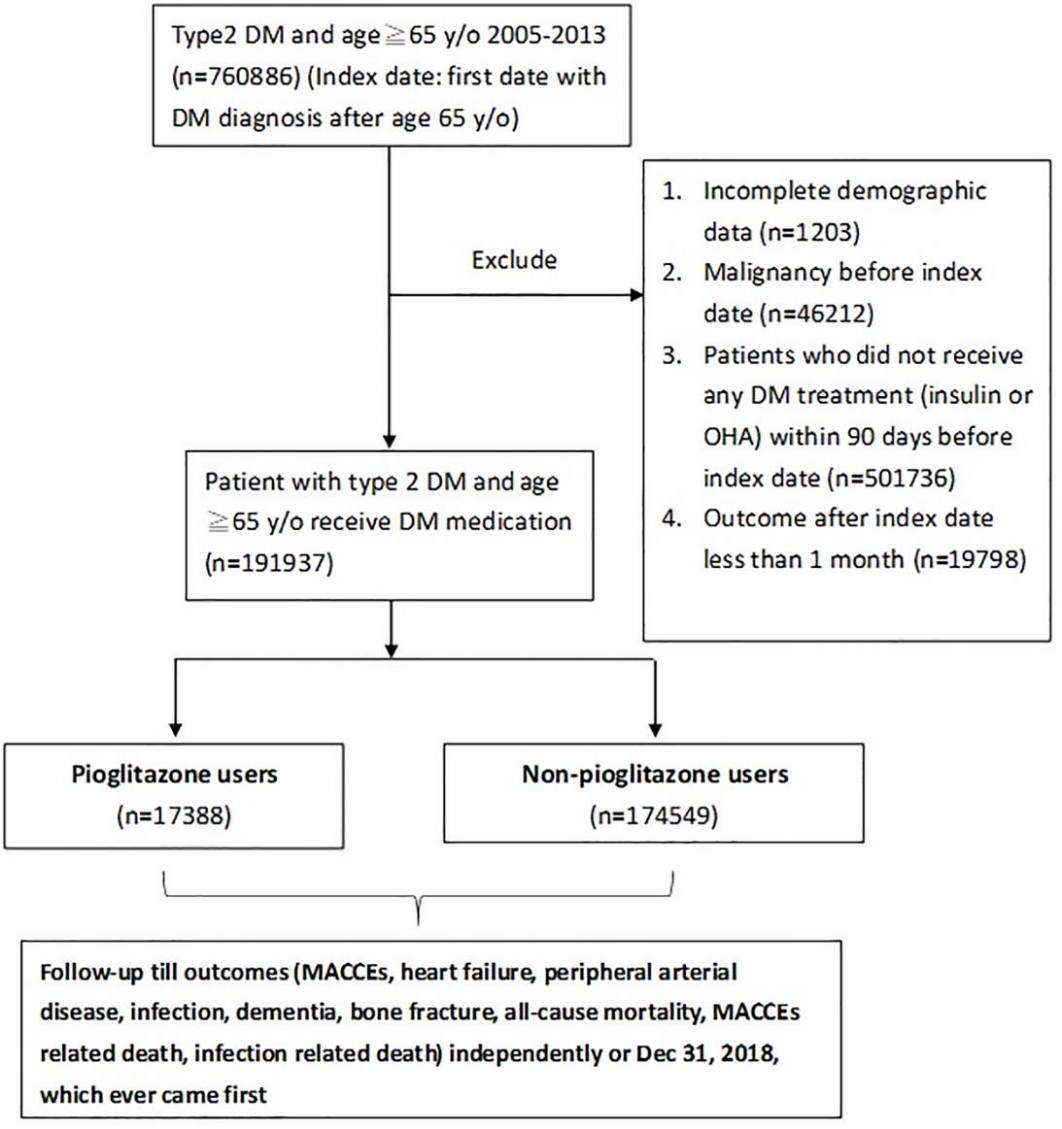
**Flowchart for the inclusion and follow-up of study patients.** Abbreviations; DM: diabetes mellitus; MACCE: major cardiac and cerebrovascular events; OHA: oral hypoglycemic agents.

As illustrated in Figure 1, type 2 diabetes patients aged ≥65 years were identified from the NHIRD. The first outpatient visit date indicating a diagnosis of type 2 diabetes, irrespective of newly diagnosed diabetes or chronic diabetes, after 65 years of age was defined as the index date. For example, a patient was diagnosed with diabetes at the age of 62 and had regular outpatient visits afterward. His index date in this study would be the date of first outpatient visit for diabetes after age 65. Patients were excluded from this study if they had the following conditions: (1) incomplete demographic data, (2) diagnosis of malignancy before the index date, (3) did not receive any OHA or insulin treatment within 3 months preceding the index date, and (4) outcomes within 1 month after the index date. The remaining patients were further divided into two groups, namely pioglitazone and non-pioglitazone groups, depending on whether they used pioglitazone within 3 months preceding the index date.

### Covariates and outcomes

The covariates in this research were age, sex, income level, Charlson Comorbidity Index (CCI) [18], place of residence, comorbidities, history of hospitalization events, and medication. Comorbidities were identified if reported for more than two outpatient visits or one inpatient stay within the year before the index date. The history of hospitalization events was tracked three years before the index date. The diagnoses of comorbidities and events during hospitalization have mostly been validated in previous NHIRD studies [19, 20]. Medications were identified following the prescriptions within 90 days before the index date.

The clinical outcomes in this study were major adverse cardiac and cerebrovascular events (MACCE) (a composite of myocardial infarction (MI), cardiogenic shock, new-diagnosis heart failure, coronary revascularization, malignant arrhythmia, and cerebrovascular events), infection, new- diagnosis dementia (all kinds included), new- diagnosis non-traffic accident bone fracture, new- diagnosis heart failure, all-cause mortality, MACCE-related mortality, and infection-related mortality. MACCE and infection were identified according to the principal diagnosis at inpatient, outpatient, or emergency room visits. New-diagnosis dementia, new-diagnosis non-traffic accident bone fracture, and new-diagnosis heart failure were identified according to the first three diagnoses at hospitalization, emergency room, or outpatient visits. In previous NHIRD validation researches, the positive predictive values for acute myocardial infarction [19], ischemic stroke [20], and CV death [21] were 88%, 88–94%, and 95%, respectively. Because dementia and heart failure are chronic diseases and the diagnosis of bone fracture may be long-term used during outpatient visits for subsequent treatment, those who have already been diagnosed with dementia, heart failure, and bone fracture before the index date would not be counted in the outcomes of new-diagnosis heart failure, new-diagnosis dementia, and new-diagnosis bone fracture. All-cause mortality was determined based on the patient’s appearance in the Taiwan Death Registry. The reason for death was determined using either the principal diagnosis in discharge records for inpatient hospital deaths or, according to the Taiwan Death Registry for outpatient deaths. The infection-related death was identified if the principal discharge diagnosis for death was infection (ICD-9-CM and ICD-10-CM codes for infection were listed in Supplementary Table 1). Because the treatment of diabetes may change from time to time and an observational study cannot guarantee an extended treatment duration of pioglitazone, this study thus only compared outcomes within five years. The follow-up time was from the index date to the first occurrence of any study outcome independently or until five years after the index date, whichever occurred first.

### Statistical analysis

We used propensity score stabilized weights (PSSWs) to balance the baseline characteristics between the two study groups [22]. The advantage of PSSWs is their ability to determine the average treatment effect for the population while maintaining the original sample size and the designated type I error [23]. We used the generalized boosted model (GBM) to obtain PSSWs for the two study groups. The GBM can automatically include interactions or polynomial terms of covariates to obtain the optimal balance between the two study groups [24]. All covariates in Table 1, except for CCI, were included in the GBM because CCI was a combination of comorbidities. We used the absolute standardized mean difference (ASMD) to examine the balance of baseline characteristics between the two study groups. An ASMD ≤0.1 indicated an insignificant difference in baseline characteristics between the two study groups [25].

**Table 1:** Demographics, comorbidities, hospitalization histories, and medication use at index date among patients older than 65 with type 2 diabetes mellitus.

	**Before PSSW**			**After PSSW**		
	**Pioglitazone**	**Non_Pioglitazone**	**ASMD**	**Pioglitazone**	**Non_Pioglitazone**	**ASMD**
	**(*n* = 17388)**	**(*n* = 174549)**		**(*n* = 16730.1)**	**(*n* = 174490)**	
Age (yrs)						
Mean	65.23 ± 1.60	66.15 ± 3.67	0.3247	65.95 ± 3.29	66.08 ± 3.55	0.0367
<75	17241 (99.15%)	165754 (94.96%)	0.7405	16002.7 (95.65%)	166355 (95.34%)	0.0204
≥75	147 (0.85%)	8795 (5.04%)		727.353 (4.35%)	8135.16 (4.66%)	
Sex						
Male	8213 (47.23%)	81330 (46.59%)	0.0128	7866.73 (47.02%)	81415 (46.66%)	0.0073
Female	9175 (52.77%)	93219 (53.41%)		8863.35 (52.98%)	93075.2 (53.34%)	
Income (NTD)						
≥25,000	1357 (7.8%)	11941 (6.84%)	0.0744	1169.56 (6.99%)	12088 (6.93%)	0.0238
15,000–25,000	4409 (25.36%)	49161 (28.16%)		4568.51 (27.31%)	48701.7 (27.91%)	
<15,000 or dependent	11622 (66.84%)	113447 (64.99%)		10992.01 (65.7%)	113700.6 (65.16%)	
Place of residence						
Urban	4914 (28.26%)	43250 (24.78%)	0.0912	4268.97 (25.52%)	43793.4 (25.1%)	0.0663
Suburban	4627 (26.61%)	46447 (26.61%)		4472.62 (26.73%)	46431.7 (26.61%)	
Rural	7390 (42.5%)	78961 (45.24%)		7474.96 (44.68%)	78495.6 (44.99%)	
Missing	457 (2.63%)	5891 (3.37%)		513.532 (3.07%)	5769.57 (3.31%)	
DM duration (yrs)						
Mean ± SD	9.34 ± 3.46	6.54 ± 4.08	0.7405	6.87 ± 3.99	6.79 ± 4.1	0.0204
Charlson’s score						
0	11834 (68.06%)	107684 (61.69%)	0.2515	10750.1 (64.26%)	108182 (62%)	0.0504
1	4336 (24.94%)	48792 (27.95%)		4490.86 (26.84%)	48556.2 (27.83%)	
2	913 (5.25%)	12734 (7.3%)		1011.43 (6.05%)	12567.1 (7.2%)	
3	213 (1.22%)	3533 (2.02%)		305.423 (1.83%)	3443.74 (1.97%)	
4	54 (0.31%)	1100 (0.63%)		91.7618 (0.55%)	1065.35 (0.61%)	
5+	38 (0.22%)	706 (0.41%)		80.46257 (0.49%)	676.13023 (0.38%)	
Comorbidities (within 1 year before index date)						
Hypertension	12069 (69.4%)	118294 (67.8%)	0.0353	11332.34 (67.7%)	118518.03 (67.9%)	0.0040
Dyslipidemia	9591 (55.2%)	77208 (44.2%)	0.2198	7745.09 (46.3%)	78898.28 (45.2%)	0.0218
Liver cirrhosis	138 (0.79%)	2576 (1.48%)	0.0644	226.45 (1.35%)	2468.78 (1.41%)	0.0053
Connective tissue disease	608 (3.5%)	6955 (3.98%)	0.0257	632.05 (3.78%)	6875.57 (3.94%)	0.0085
Atrial fibrillation	135 (0.78%)	2658 (1.52%)	0.0701	218.42 (1.31%)	2541.12 (1.46%)	0.0130
PAD	274 (1.58%)	2598 (1.49%)	0.0071	250.21 (1.5%)	2605.36 (1.49%)	0.0002
Dementia	52 (0.3%)	907 (0.52%)	0.0346	70.36 (0.42%)	871.73 (0.5%)	0.0118
Advanced CKD or ESRD	7060 (40.6%)	52125 (29.9%)	0.2263	5159.71 (30.8%)	53793.64 (30.8%)	0.0003
Bone fracture	3185 (18.3%)	23366 (13.4%)	0.1353	2369.7 (14.2%)	24128.69 (13.8%)	0.0098
Hospitalization history (within 3 years before index date)						
Heart failure	70 (0.4%)	969 (0.56%)	0.0221	79.6 (0.48%)	944.89 (0.54%)	0.0093
Myocardial infarction	84 (0.48%)	1387 (0.79%)	0.0391	94.3 (0.56%)	1337.96 (0.77%)	0.0252
Stroke	542 (3.12%)	8574 (4.91%)	0.0915	810.44 (4.84%)	8291.48 (4.75%)	0.0044
Infection	1157 (6.65%)	12988 (7.44%)	0.0307	1194.91 (7.14%)	12856.14 (7.37%)	0.0088
Medication use (within 90 days before index date)						
ACEi or ARB	10060 (57.9%)	84460 (48.4%)	0.1906	8457.67 (50.6%)	85930.65 (49.2%)	0.0264
beta-blocker	4383 (25.2%)	45923 (26.3%)	0.0252	4419.91 (26.4%)	45734.36 (26.2%)	0.0048
CCB	7107 (40.9%)	72792 (41.7%)	0.0169	6954.77 (41.6%)	72639.91 (41.6%)	0.0012
Diuretics	5169 (29.7%)	46417 (26.6%)	0.0697	4403.39 (26.3%)	46875.28 (26.9%)	0.0124
aspirin or clopidogrel	5474 (31.5%)	51582 (29.6%)	0.0419	5033.74 (30.1%)	51866.3 (29.7%)	0.0080
other NSAIDs	9876 (56.8%)	99163 (56.8%)	0.0003	9487.74 (56.7%)	99135.76 (56.8%)	0.0021
Insulin	226 (1.3%)	4871 (2.79%)	0.1055	413.49 (2.47%)	4635.13 (2.66%)	0.0118
Statins	6707 (38.6%)	47773 (27.4%)	0.2400	4809.3 (28.7%)	49532.66 (28.4%)	0.0080
fibrate or gemfibrozil	1318 (7.58%)	12925 (7.4%)	0.0067	1274.44 (7.62%)	12946.38 (7.42%)	0.0076
Metformin	4528 (26%)	52392 (30%)	0.0886	4966.7 (29.7%)	51737.26 (29.7%)	0.0008
DPP4	1539 (8.85%)	11669 (6.69%)	0.0810	1183.37 (7.07%)	12010.32 (6.88%)	0.0075
Sulfonylurea	12561 (72.2%)	117435 (67.3%)	0.1082	11321.61 (67.7%)	118187.37 (67.7%)	0.0013
Acarbose	240 (1.38%)	924 (0.53%)	0.0876	92.1 (0.55%)	1046.47 (0.6%)	0.0066
Meglitinides	1317 (7.57%)	11247 (6.44%)	0.0443	1095.57 (6.55%)	11418.09 (6.54%)	0.0002
Number of DM drugs categories						
1	12273 (70.59%)	130850 (74.97%)	0.0961	12242.8 (73.18%)	130239.7 (74.64%)	0.0227
2	4702 (27.04%)	40635 (23.28%)		4208.2 (25.15%)	41068 (23.54%)	
3	401 (2.31%)	2999 (1.72%)		273.928 (1.64%)	3113.49 (1.78%)	
4+	12 (0.06%)	65 (0.03%)		5.15475 (0.03%)	69.28949 (0.04%)	

Abbreviations: ACEi: angiotensin-converting enzyme inhibitor; ARB: angiotensin receptor blocker; ASMD: absolute standardized mean difference; CCB: calcium channel blocker; CKD: chronic kidney disease; DPP4i: dipeptidyl peptidase 4 inhibitors; ESRD: end-stage renal disease; NSAID: non-steroidal anti-inflammatory drug; PAD: peripheral arterial disease; PSSW: propensity score stabilized weighting analysis.

The incidence rates of the clinical outcomes were the total number of study outcomes during the follow-up period divided by person-years at risk. The cumulative incidence rates of the clinical outcomes versus follow-up years were plotted using the Kaplan-Meier method and compared using the log-rank test. A Cox proportional hazards model was used to obtain pioglitazone’s hazard ratio (HR) and confidence interval (CI) relative to the non-pioglitazone group. Subgroup analysis with a forest plot was performed to examine whether the benefits or risks of pioglitazone were maintained under different conditions when compared with the non-pioglitazone group. We performed PSSWs for each subgroup analysis to ensure a good balance between the baseline characteristics of the two study groups. The significance level of this study was set at 0.05.

## RESULTS

### Patient characteristics

Between 2005 and 2013, 191,937 type 2 diabetes patients aged ≥ 65 years who received at least one type of type 2 diabetes medicine were extracted from the NHIRD. Of these, 17,388 were treated with pioglitazone within 90 days before the index date (pioglitazone group), while the other 174,549 patients did not (non-pioglitazone group) (Figure 1). The demographic, comorbidity, distribution of Charlson’s score, hospitalization history, diabetes duration, and medication characteristics at baseline between the two groups are displayed in Table 1. Before PSSWs, the pioglitazone group exhibited the following characteristics: younger age, more dyslipidemia, advanced CKD or end-stage renal disease (ESRD), and more prescriptions for statins. After PSSWs, most of the ASMD values were <0.1, suggesting well-balanced baseline characteristics between the two study groups (Table 1).

### Outcomes

**Figure 2:**
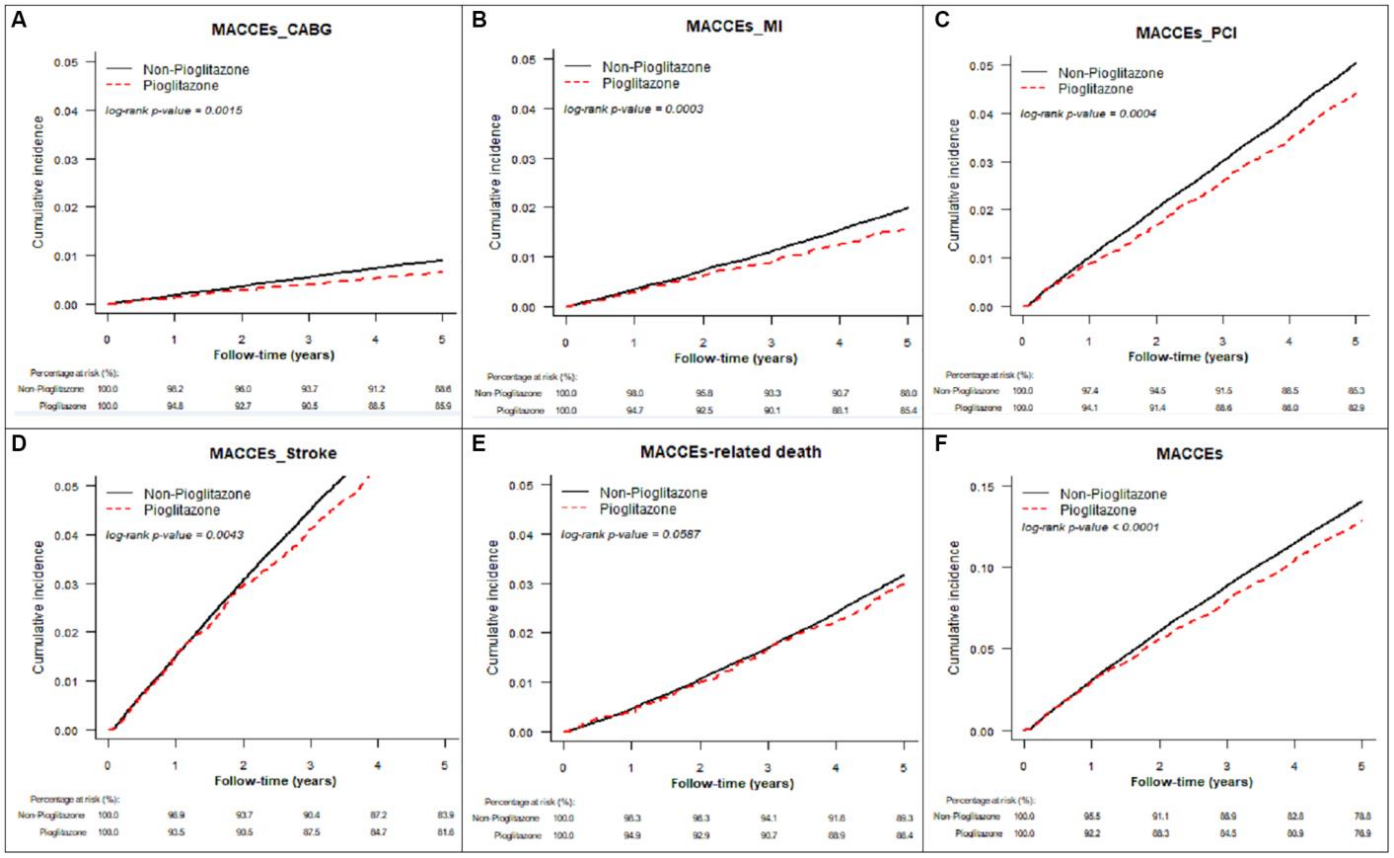
**Cumulative incidence for varied major advanced cardiac and cardiovascular events (MACCE) after propensity score stabilizing weighting.** (**A**) Coronary artery bypass graft (CABG), (**B**) Myocardial infarction (MI), (**C**) Percutaneous coronary intervention (PCI), (**D**) stroke, (**E**) MACCE-related death, (**F**) overall MACCE.

**Figure 3:**
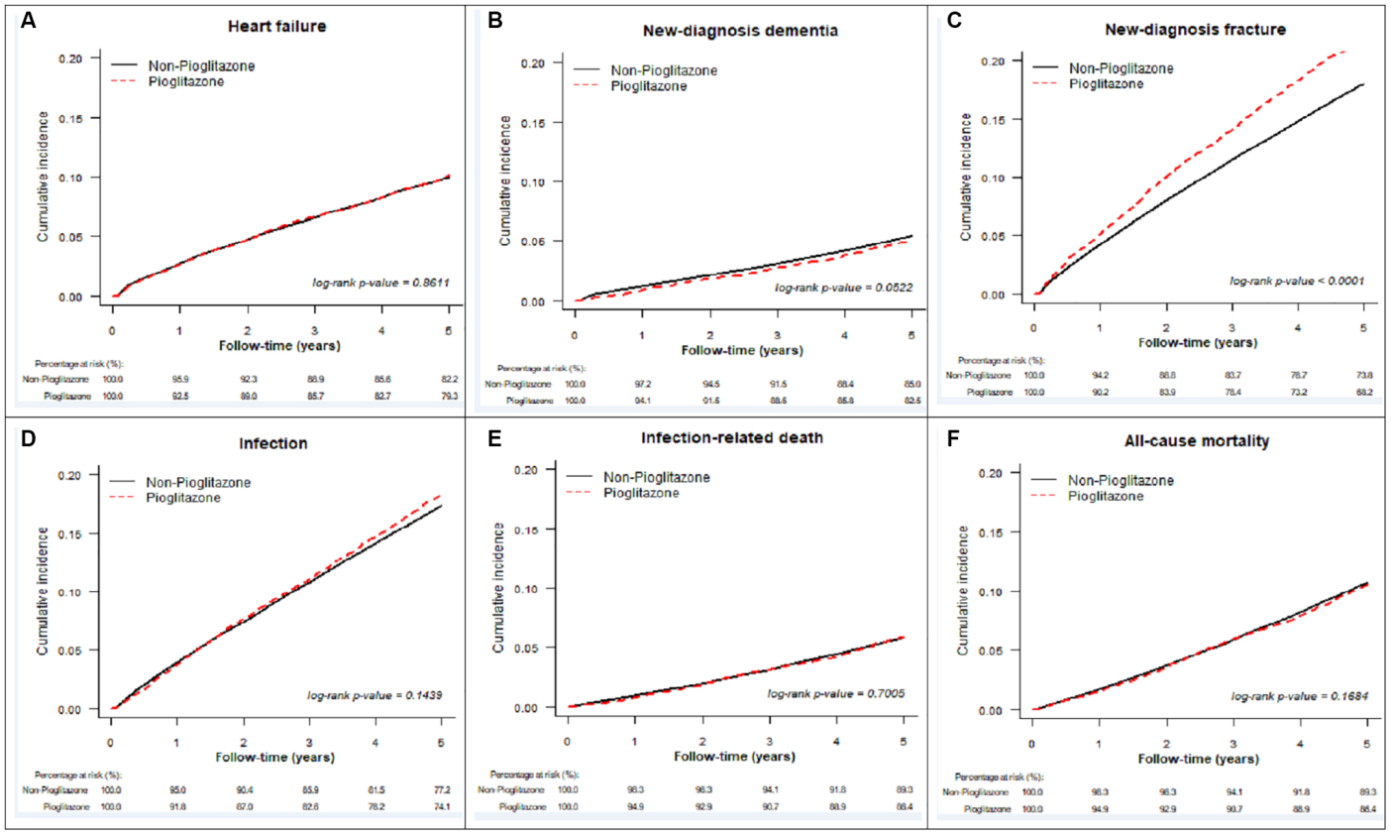
**Cumulative incidence for other study outcomes after propensity score stabilizing weighting.** (**A**) new-diagnosis heart failure, (**B**) new-diagnosis dementia, (**C**) new-diagnosis fracture, (**D**) infection, (**E**) infection-related death, and (**F**) all-cause mortality.

Table 2 represents the study outcomes after a 5-year follow-up, after PSSWs, the pioglitazone group exhibited a lower rate (per person-years) of MACCE (2.76% vs. 3.03%, hazard ratio [HR]: 0.91, 95% confidence interval [CI]: 0.87–0.95), acute myocardial infarction (0.37% vs. 0.46%, HR:0.79, 95% CI: 0.70–0.90), stroke (1.56% vs. 1.72%, HR:0.91, 95% CI: 0.86–0.97), and new-diagnosis dementia (1.32% vs. 1.46%, HR:0.91, 95% CI: 0.84–0.98) compared to the non-pioglitazone group. For coronary revascularisations, the pioglitazone group exhibited a lower rate of coronary artery bypass graft (0.16% vs. 0.21%, HR: 0.72, 95% CI: 0.59–0.88) and percutaneous coronary intervention (1.03% vs. 1.17%, HR: 0.87, 95% CI: 0.81–0.94) compared to non-pioglitazone group. However, the pioglitazone group exhibited a higher rate of new-diagnosis non-traffic-accident bone fractures (5.37% vs. 4.47%, HR: 1.24, 95% CI: 1.19–1.28). The cumulative incidence curves for study outcomes are shown in Figure 2 (varied major advanced cardiac and cardiovascular events after PSSW) and Figure 3 (other study outcomes after PSSW).

**Table 2:** Clinical outcomes of patients older than 65 years with type 2 diabetes mellitus, after propensity score stabilized weights.

**Outcome**	**Pioglitazone (*n* = 16730.1)**	**Non-pioglitazone (*n* = 174490)**	**HR (95% CI)**	***p*-value**
	**Event (Incidence, per 100 person-years)**			
MACCE_CABG	131.32 (0.16)	1844.33 (0.21)	0.72 (0.59–0.88)	0.0000
MACCE_AMI	308.94 (0.37)	4110.03 (0.46)	0.79 (0.70–0.90)	0.0000
MACCE_PCI	847.65 (1.03)	10276.31 (1.17)	0.87 (0.81–0.94)	0.0000
MACCE_Stroke	1276.19 (1.56)	14942.54 (1.72)	0.91 (0.86–0.97)	0.0000
MACCE related death	623.47 (0.74)	7723.8 (0.85)	0.91 (0.83–1.00)	0.0600
MACCE^a^	2074.67 (2.76)	23565.03 (3.03)	0.91 (0.87–0.95)	0.0000
Heart failure	2186.06 (2.77)	24037.66 (2.86)	1.01 (0.96–1.06)	0.8100
New-diagnosis dementia	1083.70 (1.32)	12776.24 (1.46)	0.91 (0.84–0.98)	0.0100
New- diagnosis fracture	3967.46 (5.37)	36043.9 (4.47)	1.24 (1.19–1.28)	0.0000
Infection	3499.31 (4.53)	37302.6 (4.53)	1.04 (1.00–1.08)	0.0400
Infection related death	1189.23 (1.40)	13588.7 (1.50)	0.98 (0.92–1.05)	0.6100
All-cause mortality	2140.48 (2.53)	24770.19 (2.74)	0.95 (0.91–1.00)	0.0700

^a^MACCE: major advanced cardiovascular events, any coronary artery bypass graft (CABG), myocardial infarction (MI), percutaneous coronary intervention (PCI), cardiogenic shock, new-diagnosis heart failure, coronary revascularization, malignant arrhythmia, or cerebrovascular events; Abbreviations: CI: confidence interval; HR: hazard ratio (pioglitazone vs. non-pioglitazone) and non-pioglitazone was the reference group).

### Subgroup analysis

**Figure 4:**
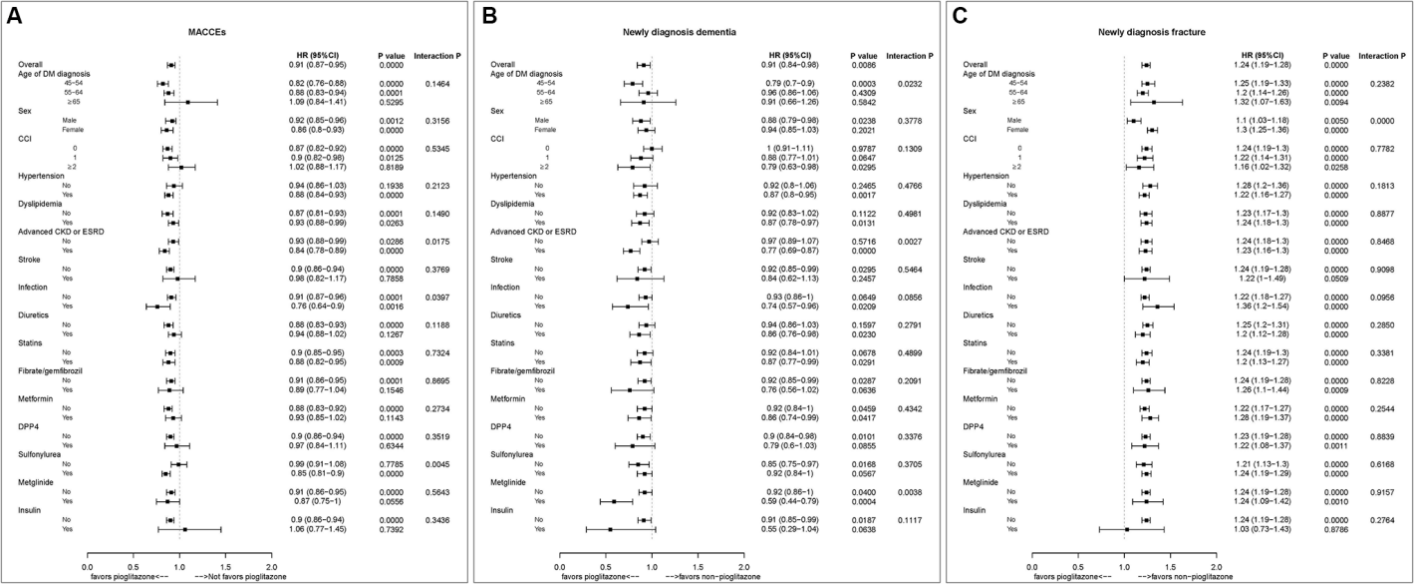
Subgroup analysis of (**A**) major cardiac and cerebrovascular events (MACCE), (**B**) new-diagnosis dementia, and (**C**) new-diagnosis fracture. Abbreviations: CI: confidence interval; HR: hazard ratio.

To determine whether the benefits or risks of pioglitazone were maintained under different conditions, we performed subgroup analyses for MACCE, dementia, and bone fractures (Figure 4). Regarding MACCE, the benefit of pioglitazone seemed to be more pronounced in patients with comorbid chronic kidney disease. Regarding new-diagnosis dementia, the benefit of pioglitazone seems to be more apparent in patients with comorbid chronic kidney disease. Lastly, regarding new-onset bone fractures, the risk of pioglitazone seems to be higher in women.

## DISCUSSION

Recently, type 2 diabetes treatment has focused on agents belonging to two newfound anti-diabetes classes, namely sodium-glucose cotransporter 2 (SGLT2) inhibitors, and glucagon-like peptide-1 (GLP-1) agonists, because of their impressive benefits in reducing the risk of CV events and heart failure [26]. However, since insulin treatment should be avoided while treating elderly type 2 diabetes patients to reduce the risks of hypoglycemia, a combination of SGLT2 inhibitors, GLP-1 agonists, and other kinds of OHA might be necessary to reach appropriate sugar levels. The lowest risk of hypoglycemia and insulin-sensitizing effect seen with pioglitazone makes it an attractive option for treating elderly type 2 diabetes patients, regardless of whether they are combined with new-generation OHA.

Because of increased oxidative stress and myocardial degeneration, the risk of CVD increases, and CV event is the leading cause of death in the elderly population [27, 28]. Several previous studies, including randomized controlled trials (RCTs) such as the PROactive study and PERISCOPE trial [29, 30], as well as high-quality observational studies [31], have indicated that the use of pioglitazone can reduce CV risk. However, these previous studies did not focus on elderly patients. This study demonstrated that using pioglitazone was associated with a significant 9% reduction in the probability of MACCE in the elderly type 2 diabetes population. Regarding the reduction of MACCE risks, the beneficial effect of pioglitazone in this study is not as much as in previous RCTs. For example, in the PROactive study, pioglitazone was associated with a significant 16% reduction in the risk of MACCE, and in the IRIS study, using pioglitazone also significantly reduced the risk of stroke or myocardial infarction by 24%. In this regard, because the benefit of pioglitazone on cardiovascular outcomes mainly resulted from the retardation of atherosclerosis progression, which is a time-consuming process, a more extended treatment period may be required to determine the differences. Interestingly, the reduction of MACCE in the pioglitazone group was mainly attributed to the lower risks of coronary artery diseases and coronary artery revascularisations in this study. Thus, for elderly patients with diabetes, pioglitazone might be an appropriate option if the first priority is reducing coronary artery disease risks. In addition to MACCE, the effect of pioglitazone on the risk of incident dementia was another interest of this study. Dementia is one of the most crucial health issues worldwide in the elderly population [32]. Because of the prolonged disease course and progressive health care requirement for patients with dementia, their average Medicare expenditures are 57% greater than other chronic diseases [33], thus making dementia a social problem due to its considerable costs. Diabetes has been recognized as a crucial risk factor for dementia in elderly adults [34]. Insulin resistance in the brain and diabetes-associated vascular dysfunction may underlie the association between diabetes and dementia [35]. Pioglitazone, an insulin sensitizer, should have a role in treating dementia. Previous studies have shown that pioglitazone can retard brain aging and improved cognitive function [36]. In this study, elderly diabetes patients under pioglitazone had a 9% reduction in the risk of incident dementia. Although the incidence of dementia between users and non-users of pioglitazone is not substantially different, considering the long duration of dementia and the substantial medical costs, any slight reduction in incident dementia could lead to decreased expenses for the healthcare system.

Regarding drug safety, because of sodium reabsorption and peripheral vasodilatation [37], new-onset heart failure is of greatest concern when using pioglitazone. In a previous study, our research group proved that using pioglitazone does not increase the probability of new-onset heart failure or the requirement for dialysis in the advanced CKD population [38], which is a susceptible group for fluid accumulation. This study also demonstrated that using pioglitazone did not increase the probability of heart failure in the elderly diabetes population. According to these results from different susceptible populations, although using pioglitazone may occasionally induce peripheral edema, it does not increase the risk of severe complications of fluid accumulation. However, this study raised concerns about bone fractures when pioglitazone was used. Previous RCTs enrolling general diabetes or non-diabetes patients with a history of transient ischemic attack have demonstrated a possible association between the use of TZD and subsequent bone fracture, especially among postmenopausal women [39]. Although not fully elucidated, preclinical data indicated that PPAR-γ activation might decrease bone mass by inhibiting the bone formation and stimulating bone resorption [40]. Similarly, through real-world data, this study exhibited a 24% increase in relative bone fracture risk using pioglitazone in the elderly type 2 diabetes population, especially among female patients. Thus, clinicians need to balance the risks of bone fracture and the benefits of prescribing pioglitazone when treating elderly patients with diabetes. For elderly patients already receiving pioglitazone, falls prevention and regular screening for bone health are necessary.

This study had several limitations that should be acknowledged. First, some important information, including glycohemoglobin levels, serum sugar levels, creatinine, lipid profile, albumin, proteinuria, body mass index, heart function, frailty scale, and blood pressure control, were unavailable in the NHIRD. Although we have well-matched different kinds of diabetes medications, the total number of diabetes medications, and the duration since the first diagnosis of diabetes in this study, we cannot confirm the equal sugar control between pioglitazone users and non-users. Second, even after PSSW, which included the most relevant confounders to our knowledge, there might have been some residual bias due to the observational study. Third, the effect of the combination of pioglitazone and new-generation OHA, such as SGLT2 inhibitors and GLP-1 agonists, was not analyzed because the National Healthcare Insurance of Taiwan reimbursed these DM medications after 2017.

In conclusion, using pioglitazone may reduce the risks of several crucial outcomes, MACCE, and dementia, without increasing the risk of heart failure in the elderly DM population. However, it would increase the probability of bone fractures, especially in female patients. Although the results of this study were roughly similar to that in previous research among the general population, this study first evaluated the role of pioglitazone in elderly diabetes patients, a particularly vulnerable population. This study especially investigated the influence of pioglitazone on the risks of dementia and bone fracture, which are more crucial for the elderly than for middle-aged patients. This information may help clinicians choose appropriate agents to treat elderly patients with DM and prevent possible complications in advance.

## Supplementary Materials

Supplementary Table 1
