# Supplementary material for: Pioglitazone reduces cardiovascular events and dementia but increases bone fracture in elderly patients with type 2 diabetes mellitus: a national cohort study
Source: Aging (Albany NY). 2023 Apr 7;15(7):2721–33. doi: 10.18632/aging.204643 (PMC10120904; doi:10.18632/aging.204643)
Supplement: Supplementary Table 1 [file aging-15-204643-s001.pdf]

SUPPLEMENTARY TABLE

Supplementary Table 1. Disease or procedure codes for outcomes in this study.

Disease	ICD-9-CM	ICD-10-CM
CABG	68023A, 68023B, 68024A, 68024B, 68025A, 68025B	02100, 02110,02120, 02130
AMI	410	I21, I22
PCI	33076A, 33076B, 33077A, 33077B, 33078A, 33078B	02103,02104, 02113, 02114, 02123, 02124, 02133, 02134
Stroke	430-437	I60-64, G45.0, G45.1, G45.4, G45.8, I67
Heart failure	428	I50
Dementia	290.0-290.4, 294.1, 331.0,331.1-331.2	F00-F03
Non-traumatic bone fracture	733.1, 805-829, exclude E810-E819	M484, M495, M80, M843-844, M907, M966, S02, S12, S22, S32, S42, S52, S62, S72, S82, S92, T02, T08, T10, T12, T142<br>Exclude E810-E819
**Infection**		
Sepsis	038, 995.91, 995.92, 020.2, 785.52, 790.7	R65.20, R65.21, R78.81, A41.9, A49.9, B96.89
Pneumonia	481-486 (exclude 484)	J14, J15, J16, J17, J18, J12, J69, J95.8, J09, M96, B25.0, B96.1, B95.3, B37.1, B38.0, A74, A40.3, A02.22, B45.0, B44.9, O89.01
Empyema	510.0, 510.9	J85, J86
Cellulitis	681, 682	L03, H60, H05, H00, K61
Necrotizing fasciitis	728.86	M72.6
Urinary tract infection	590, 595.0, 599.0	N39.0, N30.01, N30.90, N99.51x, B37.41, A54.01, A56.01, N10.xx, N11.xx, N12.xx, N15.xx, N16.xx, T83.511
Biliary tract infection	576.1, 575.0, 574.00	K83
Brain abscess	324	G06, A54.82, B43.1, A06.6
Liver abscess	572.0	K75
Perianal abscess	566	K61
Bacterial meningitis	320	G03
Septic arthritis	711	M00, M19.90
Infection of catheter, device, implant, and graft	996.6, 999.3	T80.2, T82.7, T85.7, T83.51,
Peritoneal and retroperitoneal infection	567	K65, K67, A18.31, T85.71, N73, K68.11
Osteomyelitis	730.3, 730.8, 730.9	M86, M46.2-M46.5
Infective endocarditis	421	I33, I38

Abbreviations: AMI: acute myocardial infarction; CABG: coronary artery bypass graft; PCI: percutaneous coronary intervention.
